# A Combinatorial Protein Microarray for Probing Materials Interaction with Pancreatic Islet Cell Populations

**DOI:** 10.3390/microarrays5030021

**Published:** 2016-08-10

**Authors:** Bahman Delalat, Darling M. Rojas-Canales, Soraya Rasi Ghaemi, Michaela Waibel, Frances J. Harding, Daniella Penko, Christopher J. Drogemuller, Thomas Loudovaris, Patrick T. H. Coates, Nicolas H. Voelcker

**Affiliations:** 1 Australian Research Council Centre of Excellence in Convergent Bio-Nano Science and Technology, Future Industries Institute, University of South Australia, Adelaide 5095 SA, Australia; Bahman.Delalat@unisa.edu.au (B.D.); rasighaemis@yahoo.com (S.R.G.); Fran.Harding@unisa.edu.au (F.J.H.); 2 School of Medicine, University of Adelaide, Adelaide5005 SA, Australia; Darling.Rojas@sa.gov.au (D.M.R.-C.); Daniella.Penko@sa.gov.au (D.P.); Chris.Drogemuller@sa.gov.au (C.J.D.); Toby.Coates@ sa.gov.au (P.T.H.C.); 3 Centre for Clinical and Experimental Transplantation, Adelaide 5000 SA, Australia; 4 Immunology and Diabetes Unit, St. Vincent’s Institute of Medical Research, Fitzroy 3065 Vic, Australia; mwaibel@svi.edu.au (M.W.); tloudovaris@svi.edu.au (T.L.); 5 Central Northern Adelaide Renal Transplantation Service, Royal Adelaide Hospital, Adelaide 5000 SA, Australia

**Keywords:** ECM proteins, microarrays, pancreatic islets, high throughput screening

## Abstract

Pancreatic islet transplantation has become a recognized therapy for insulin-dependent diabetes mellitus. During isolation from pancreatic tissue, the islet microenvironment is disrupted. The extracellular matrix (ECM) within this space not only provides structural support, but also actively signals to regulate islet survival and function. In addition, the ECM is responsible for growth factor presentation and sequestration. By designing biomaterials that recapture elements of the native islet environment, losses in islet function and number can potentially be reduced. Cell microarrays are a high throughput screening tool able to recreate a multitude of cellular niches on a single chip. Here, we present a screening methodology for identifying components that might promote islet survival. Automated fluorescence microscopy is used to rapidly identify islet derived cell interaction with ECM proteins and immobilized growth factors printed on arrays. MIN6 mouse insulinoma cells, mouse islets and, finally, human islets are progressively screened. We demonstrate the capability of the platform to identify ECM and growth factor protein candidates that support islet viability and function and reveal synergies in cell response.

## 1. Introduction

Diabetes is a serious metabolic disorder, caused by inadequate insulin secretion from pancreatic islets, and affects over 400 million people worldwide [1]. In Type 1 diabetes, autoimmune attack targets the destruction of islets and their insulin-secreting β cells [2]. In type 2 diabetes, insulin resistance of peripheral tissues can lead to chronically high blood sugar levels, placing an inordinate demand on β cells for insulin supply, eventually overwhelming the capacity of β cells to respond [3]. The high demand on β cells results in a high rate of malfunction and attrition within this cell population. Over time, high glucose levels lead to secondary damage due to chronic hyperglycemia, especially of microvascular circulation, affecting the heart, retina and kidneys [4]. Since diabetes mellitus can be regarded as a deficiency of a single cell type, it represents an attractive target for cell therapy. β cell replacement therapy is already in clinical use in the form of cadaveric islet transplantation [5,6,7]. However, the supply of islets is insufficient to meet demand, limiting this treatment to a subset of severely affected patients [8]. In addition, long-term islet graft survival rates are low [9]. Increasing islet survival prior to and during the peri-transplant period is critical to transplant success [10].

Cellular function is informed by the cell microenvironment, which incorporates a mixture of proteins and polysaccharides known as the extracellular matrix (ECM) in native tissue [11]. Although the composition and architecture of ECM varies broadly among tissues, the most basic roles of ECM are to provide physical support and sites for cellular attachment [12,13,14]. The ECM also is responsible for transmitting a multitude of chemical and mechanical signals to the cells that regulate not only cell adhesion but also other key features of cellular physiology such as proliferation, differentiation and migration [15]. The influence of ECM proteins within the islet basement and interstitial membranes on pancreas development, function and pathogenesis is increasingly being recognized [16,17,18,19]. Cell microarrays allow a large number of candidate materials to be screened in parallel [20,21,22,23,24]. Biomolecules of interest are printed as microscale spots onto a glass or silicon substrate, while the rest of supportive substrate is coated to resist non-specific cell, making every printed spot an independent experimental replicate [21,25]. This study investigates the contribution of ECM proteins and growth factors towards β cell attachment and insulin expression as a marker of β cell function. We present a cell microarray platform to examine β cell and islet interactions with various protein candidates for encapsulation matrices in a high throughput manner. A microarrayed library with 201 different protein combinations was printed and used to examine attachment and insulin expression of insulinoma cells as well as mouse and human islets. Several proteins supportive of islet attachment to a substrate were identified.

## 2. Materials and Methods

### 2.1. Protein Microarray Fabrication

Plasma polymerization was performed to deposit a thin polymer coating on glass slides [26]. Briefly, glass slides were placed into the chamber of a custom-built plasma reactor. The plasma chamber was pumped down to its base pressure of approximately 30 mTorr. Afterwards, the inlet valve was gradually opened to allow air to flow into the chamber and to stabilize the pressure at 200 mTorr. Subsequently, an air plasma with a RF frequency of 13.56 MHz, at a vapor pressure of 200 mTorr, input power of 50 W and treatment time of 1 min was used to clean the surface of the slides in order to ensure proper bonding of the subsequent plasma polymer layers to the glass substrate, followed by the evacuation of the plasma chamber back down to its base pressure of 30 mTorr. Hexamethyldisiloxane (HMDSO) (Sigma–Aldrich, St. Louis, MO, USA) vapors were introduced into the plasma chamber and the pressure was observed to be stable at 200 mTorr for at least 1 min. This was followed by plasma polymerization with a RF frequency of 13.56 MHz, at a vapor pressure of 200 mTorr, input power of 25 W and deposition time of 1 min. The chamber was pumped down to its base pressure and the input monomer changed to allyl glycidyl ether (AGE) (Sigma–Aldrich). Plasma polymerization was performed using AGE as the monomer at a pressure of 200 mTorr at 25 W input power for 1 min in constant wave mode, then 2 min in pulsed mode (20 ms off, 1 ms on).

To provide islets with surrogate materials that furnish the same chemical signals that preserve cell function in native tissue, ECM and growth factors were printed on the cell microarrays [27]. These included type I collagen (Col I) (Millipore, Bedford, MA, USA), type II collagen (Col II) (Sigma–Aldrich), type III collagen (Col III) (Sigma–Aldrich), type IV collagen (Col IV) (Sigma–Aldrich), laminin 111 (Ln) (Sigma–Aldrich), fibronectin (Fn) (Sigma–Aldrich), fibroblast growth factor 2 (FGF-2) (Peprotech, Rocky Hill, NJ, USA), Insulin-like growth factor 2 (IGF-2) (R & D Systems, Minneapolis, MN, USA), vascular endothelial growth factor (VEGF) (Sigma–Aldrich), exenatide (Exen) (Amylin Pharmaceuticals, San Diego, CA, USA) and vitronectin (Vn) (Stem Cell Technologies, Vancouver, BC, Canada). Briefly, all proteins used for printing were dissolved in Dulbecco’s modified phosphate buffered saline (dPBS, Sigma–Aldrich). The protein solutions were placed into a 384-well plate. A high-precision robotic non-contact sciFLEXARRAYER S3 (Scienion, Berlin, Germany) was used to spot 10 nL volume of proteins onto epoxy plasma polymer glass slides, yielding spots about 400–450 µm in diameter, with a center-to-center spacing of 1000 μm. After printing, arrays were incubated in a humidified atmosphere for 16 h at 4 °C. To block nonspecific protein adsorption and cell attachment, the printed slides were then incubated in 5% bovine serum albumin (BSA) (Sigma–Aldrich) in sterile dPBS at pH 7 for 16 h at 37 °C. The arrayed slides were rinsed with sterile dPBS and 4 % antibiotic- antimicotic solution (Sigma–Aldrich) before use.

### 2.2. Insulinoma Cell Culture

MIN6 clone B1 mouse insulinoma cells (ATCC CRL-11506) were grown in high-glucose Dulbecco’s modified Eagle’s media (DMEM) (Sigma–Aldrich) with 2.5 mM GlutaMax (Life Technologies, Carlsbad, CA, USA) containing 15% fetal bovine serum (FBS) (Sigma–Aldrich), 100 U/mL streptomycin, 100 µg/mL penicillin (Sigma–Aldrich) and 71 µM β-mercaptoethanol (Sigma–Aldrich) freshly added. Cell cultures were maintained at 37 °C in 5% CO_2_ humidified air. The culture medium was changed twice a week and cells were replated when reaching 80%–90% confluence. MIN6 cells (1 × 10^5^ cells/mL) were seeded onto cell microarray substrates and cultured in contact with arrays at 37 °C and 5% CO_2_. Loosely attached cells were removed after 2 h by washing with prewarmed DMEM. The cells were subsequently maintained in fresh high-glucose DMEM with 2.5 mM GlutaMax containing 15% FBS, 100 U/mL streptomycin, 100 µg/mL penicillin and 71 µM β-mercaptoethanol at 37 °C in a 5% CO_2_ humidified atmosphere for up to 2 days.

### 2.3. Primary Human Islet Culture

Human islets of Langerhans (female donor, 65 years old, with body mass index (BMI) 27.5) unsuitable for clinical islet transplantation were provided by the St. Vincent’s Institute of Medical Research, Victoria, Australia under the auspices of the Australian Islet Consortium. Approval for research use of human islets was obtained from the Royal Adelaide Hospital (project 100205b, approved 29 September 2010) and University of South Australia (project 33541, approved 14 August 2014) research ethics committees. Human islets cultured in CMRL 1066 medium (Life Technologies) supplemented with 15% FBS (Life Technologies), 2 mM GlutaMax, 10 mM 4-(2-hydroxyethyl)-1-piperazine-ethanesulfonic acid (HEPES) (Sigma–Aldrich). Primary human islets (2 × 10^3^ islets/mL) were seeded onto the microarray and cultured in contact with arrays at 37 °C and 5% CO_2_. Non-bound islets were removed after 16 h by washing with prewarmed CMRL 1066 medium. The cells were subsequently maintained in fresh CMRL 1066 medium including 15% FBS at 37 °C in a 5% CO_2_ humidified atmosphere for up to 2 days.

### 2.4. Primary Mouse Islet Isolation and Culture

All experimental procedures were approved by the Animal Ethics Committee of the University of Adelaide and conform to the guidelines set out by the Australian Code of Practice for the Care and Use of Animals for Scientific Purposes. Primary pancreatic islets were isolated from 6- to 12-week-old male C57B6 mice (University of Adelaide Laboratory Animal Services, Adelaide, SA, Australia). Briefly, 3 mL cold M199 medium (Sigma–Aldrich) containing 0.67 mg collagenase (Liberase TL grade; Roche Diagnostics, GmbH, Germany) per pancreas was infused into the pancreatic duct in situ. The pancreas was removed and digested at 37 °C for 14–16 min. Islets were purified on a Ficoll gradient (GE Healthcare, Amersham, UK). Following extensive washing, islets were grown (37 °C, 5% CO_2_) in RPMI 1640 medium ( Sigma–Aldrich) supplemented with 2.5 mM GlutaMax, 100 U/mL streptomycin, 100 µg/mL penicillin, and 10% fetal calf serum for up to 2 days. Primary mouse islets (2 × 10^2^ islets/mL) were seeded onto array and cultured in contact with arrays at 37 °C and 5% CO_2_. Non-bound islets were rinsed off after 16 h by washing with prewarmed RPMI. The cells were subsequently incubated in fresh CMRL 1066 medium including 15% FBS at 37 °C and 5% CO_2_ for 2 days.

### 2.5. Fluorescence Microscopy and Cell Viability

For fluorescence microscopy, fixed cells were permeabilized with 0.3% Triton X-100 (Sigma-Aldrich) for 5 min at room temperature. Nuclei of cells were stained with 2 µg/mL Hoechst 33342 (Life Technologies) for 10 min at room temperature. Actin was stained with 100 mM Tetramethylrhodamine (TRITC)-labelled phalloidin (Sigma-Aldrich) for 45 min. Stained cells were imaged using the Operetta High Content Imaging System (PerkinElmer, Hamburg, Germany), which combines fluorescence microscopy with automated image acquisition and quantitative analysis. Images were acquired using a 20× long working distance (LWD) objective in wide-field mode in combination with appropriate filters. The insulin intensity score was calculated by the Operetta imaging system as the average pixel intensity within the microspot above a threshold fluorescence value normalized to the number of cells within the spot.

A Live/Dead cell assay was also performed by incubating cells with final concentrations of 15 µg/mL fluorescein diacetate ( Sigma-Aldrich) and 5 mM propidium iodide (Sigma-Aldrich) for 3 min at 37 °C. Cells numbers stained with each dye were enumerated by the Operetta imaging system.

### 2.6. Immunocytochemistry

To stain insulin expressing cells, the microarray slides were washed with dPBS, fixed with 4% paraformaldehyde solution for 20 min, permeabilized with 0.3% Triton X-100 in dPBS for 20 min, and then blocked with 3% goat serum in dPBS for 30 min. Primary antibody (guinea pig anti-insulin, EMD Millipore, Billerica, MA, USA) diluted in dPBS was incubated with the cells for 16 h at 4 °C. The slides were washed with dPBS, followed by incubation with a goat anti-guinea pig rhodamine conjugated antibody (Jackson ImmunoResearch, West Grove, PA, USA) diluted in dPBS incubated for 2 h at room temperature. The slides were then washed with dPBS and MilliQ water to remove the salts, and air dried. The slides were imaged and quantified with the Operetta High Content Imaging System. For quantitative analysis, images of insulin immunofluorescence were analyzed for the total brightness of cytoplasmic insulin. Images were first corrected by subtracting the average background fluorescence as determined from a non-cellular region in the images.

## 3. Results

### 3.1. Protein Microarray Fabrication

**Figure 1:**
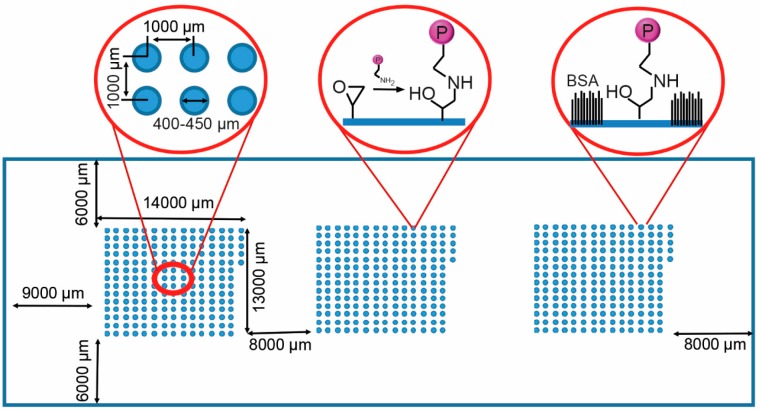
Schematic of the protein microarray on the glass slide with three blocks of 201 protein including the dimensions of the blocks and their separation. The left inset shows spot dimensions and spacing. The AGEpp coating displays epoxy groups, enabling covalent conjugation of proteins to the surface (**middle** **inset**). The non-printed surface was passivated with bovine serum albumin (BSA) (**right inset**).

For the reproducible generation of the ECM protein and growth factor microarrays, glass slides presenting epoxy functional groups for protein binding were created, in this case by coating the slides with AGE plasma polymer [27]. In this study, 11 proteins were used to create a library of 201 microspots in a combinatorial manner (Table 1). A piezoelectric arrayer was used to dispense consistent nanoliter volumes of protein solution. The layout of the microarray slide is shown in Figure 1. The candidates investigated included ECM components, Ln, Fn and Vn, the collagen isoforms Col I, Col II, Col III and Col IV, three growth factors, IGF2, FGF2, and VEGF, and the GLP-1 agonist Exen. Col I, Col II, Col III, Col IV, Ln and Fn were printed as single proteins or binary combinations at a concentration of 100 µg/mL, FGF-2, IGF-2, VEGF and Exen at a concentration of 25 µg/mL and Vn at 50 µg/mL. Combinations of three proteins were printed with one protein as the dominant component (listed first), printed at the same concentration as listed above for single and binary component spots; minor components (listed second and third) were printed at 2/3 concentration. Three array clusters, each consisting of 201 combinatorial spots, were printed. Microspots measured 400–450 μm in diameter and were spotted with a 1000 μm center-to-center distance (Figure 1). Each microarray consisted of combination of 11 different ECM proteins and growth factors. The remaining unreacted epoxy groups on the slide were then blocked with BSA to avoid non-specific cell or islet attachment in between the spots (Figure 1).

### 3.2. Profiling Cell Adhesion to Different Protein Combinations

**Figure 2:**
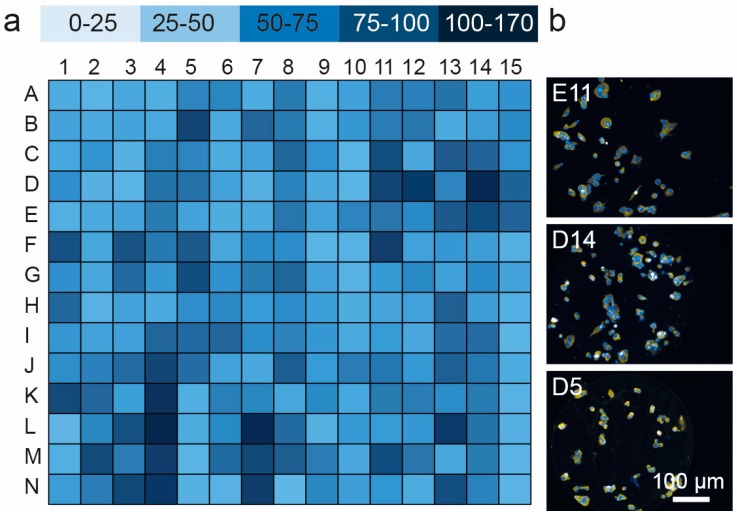
Quantification of MIN6 adhesion to the spots on protein microarray (for layout see Table 1): (**a**) map of cell attachment MIN6 cells for all of 201 protein combinations in the microarray; and (**b**) representative images of MIN6 cells adhering to protein spots (E11, Ln/Fn/Exen, D14, FGF-2/Col IV/Vn, D5, FGF-2/Vn) demonstrating selective adhesion in the locations of protein, *N* = 3.

The cell microarrays were first used to compare the adhesion of MIN6 mouse insulinoma cells to a panel of ECM proteins and growth factors. Adhesion was measured on triplicate arrays (Figure 2 and Figure S1). Triple protein combinations dominated the top 10% of candidates identified (Table 2). FGF2 was present as the major component in seven of these twenty combinations. Vn and Fn occurred most frequently (9/20 and 8/20) as minor components of these top ranked combinations. While there was no significant difference in the number of cells populating each spot on the top 10% of candidates (*p* > 0.05, ANOVA), the number of cells present on the microspots in this 10% of candidates was eight-fold higher than the bottom 10% (*p <* 0.001, *t*-test with Welch’s correction).

We then attempted to use this platform to identify protein combinations that are conducive to islet attachment. Mouse islet attachment to the library of substrates embodied in the array was probed. The islet populations were anticipated to adhere with different avidities to various combinations of ECM protein and growth factor due to their specific cell surface receptor expression, and additional three-dimensional properties of mouse and human islets. We quantified adhesion patterns by the number of nuclei visible in the focal plane. Primary mouse islet attachment was highest on spots comprised of Col IV/Col II/Fn (H6), Col IV/Col II/IGF-2 (J6), Col IV/Col II/Vn (M6), Ln/Col I/Exen (G9), FGF-2/Col I/Fn (G12) and Col IV/Col II/VEGF (K6) (Figures S2a,b and S3). These combinations achieved an average adhesion measure of >100 nuclei per spot. The combinations identified as optimal for mouse islet adhesion were distinct from those obtained for MIN6 cells, clustering in the second and third quartiles of the MIN6 dataset. In contrast to conclusions drawn from the MIN6 data, Col IV was the most prevalent protein in the highly ranked candidates. To demonstrate proof-of-concept using human islets, adhesion of a donor islet preparation was assessed using the same array format. The best primary human islet attachment (>100 cells per spot) was observed on combinations of Col IV/Col II/Ln (G6), Col IV/Col II/Fn (H6), Col IV/Col I/Vn (E6), Ln/Col II/Vn (B10), FGF-2/Col I/Exen (J12) and FGF-2/Fn/Vn (M14) (Figures S2c,d and S4). Similar to mouse islets, Col IV recurred as the major component in these highest ranked combinations.

### 3.3. Cell Viability and Insulin Synthesis by MIN6 Cells

**Figure 3:**
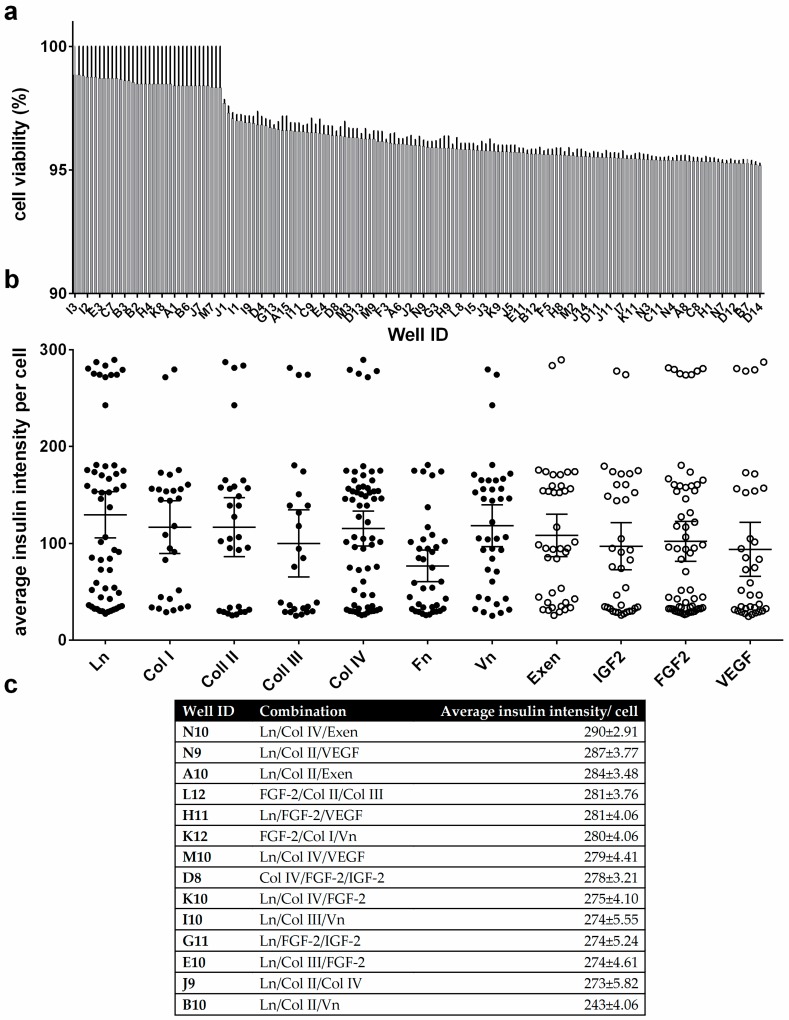
Cell viability and quantitation of cytoplasmic insulin fluorescence intensities from MIN6 cells. Only cell-populated microspots were analyzed: (**a**) Average MIN6 cell viability on the protein microarray; (**b**) Quantitation of insulin expression intensity per cell. Average insulin intensity per cell is shown for each protein tested, collating all spot combinations that include that protein. Whisker plots show the mean and 95% confidence interval of the mean for each protein; (**c**) Top combination candidates identified for insulin expression intensity per cell. All error bars represent ± standard error of the mean (SEM). *n* = 3

**Figure 4:**
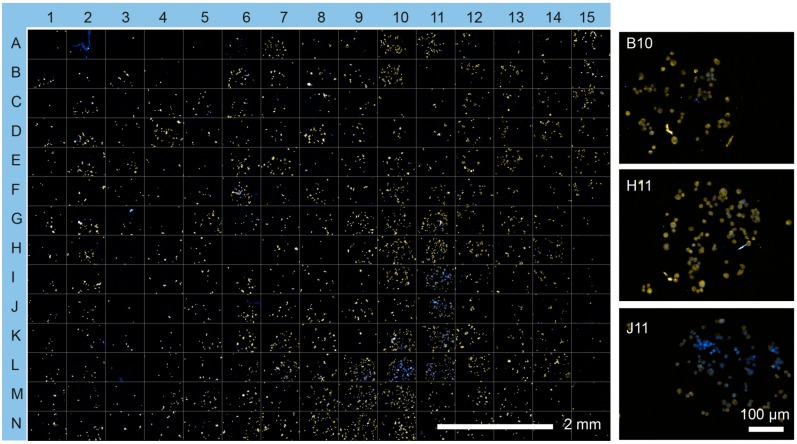
Insulin expression by MIN6 cells on the cell microarray. Insulin protein expression was visualized using immunocytochemistry (**yellow**) and counterstained with Hoechst 33342 (**blue**). Insets show selected microspots (B10, Ln/Col II/Vn; H11, Ln/FGF-2/VEGF; J11, and Ln/FGF-2/Vn) at higher resolution.

To assess basic β cell function in more detail, MIN6 cells were tested on the protein microarray to investigate cell viability. MIN6 cells showed viability of greater than 95% on all spots, as determined by propidium iodide/fluorescein diacetate staining (Figure 3a and Figure S5). To compare insulin production of MIN6 cells on the microarray spots, the cells were cultured for an additional 48 h, stained with anti-insulin antibodies and visualized by fluorescence microscopy (Figure 3b and Figure 4). Figure 4 shows that the majority of MIN6 cells on the microarray stained strongly with the anti-insulin antibody. No direct correlation was found between insulin expression and cell viability or cell number (*R*^2^ = 0.034 and 0.0073, linear regression model, Figure S6). Fourteen combinations were identified as exhibiting particularly high insulin expression (Figure 3c). These combinations produced average insulin staining intensities that were significantly greater than the rest of the candidates tested (*p =* 0.001, ANOVA/Tukey post hoc test). Notably, Ln was the dominant factor in eleven of the fourteen high expressing combinations. Thirteen combinations contained at least one collagen component and nine contained a growth factor (IGF2, FGF2 or VEGF). FGF2 featured in six of these combinations. Exen, known to activate the GLP-1 receptor, leading to an increase in insulin synthesis and release from β cells [28], was present in two of the top three candidate combinations identified. However, Fn was absent from all combinations in the high expressing set.

## 4. Discussion

With the growing and considerable research effort underway to facilitate cell therapies for the treatment of diabetes mellitus, there is an increasing need to create materials that retain β cell function and survival. One important aspect in this line of research is to investigate interactions between islets and immobilized ECM protein and growth factors to find ways to maintain and improve islet viability and function during the peri-transplant period. The influence of ECM proteins on islet viability and function is well known, and some of these proteins have been incorporated into scaffold and encapsulation materials for islet transplant [12,19,28,29]. However, previous studies have been limited in the numbers of combinations of proteins and functional peptides able to be tested. To address this issue, the microarray format described here was designed to screen ECM proteins and growth factors in a combinatorial manner, providing crucial information about islet attachment, viability and insulin secretion. Furthermore, the array was designed to allow screening of high numbers of immobilized proteins with minimal cost and time. The quantitative evaluations of cell attachment require reproducible substrate preparation cell culture procedures. The epoxy plasma polymer-coated surface in combination with microprinting was utilized to create protein microarrays. This allowed the covalent attachment of protein combinations in the form of printed microspots on the surface. Epoxy groups in between the spots were reacted with BSA to block cell attachment there.

Here we showed that microarrays are capable of differentiating β cell adhesion patterns and functional response across a library of candidate substrates [12,19,29,30]. Insulin expression correlated with Ln and collagen content. Increased insulin gene expression on both laminin and collagen substrates has been reported previously [28,29,30]. Intriguingly, while Col I, Col III and Col IV have been detected within the islet basement membrane of several species [12], Col II has not, to our knowledge, been associated with islet ECM or islet function previously. This effect may be due to integrin binding sites common between the isoforms of collagen, allowing them to substitute for one another [31]. In contrast to previous work showing an increase in insulin gene expression and release on Fn-containing substrates [29,30], Fn was notably absent from the top candidates identified for insulin expression. The addition of growth factors to ECM protein substrates appeared to further benefit MIN6 and islet adhesion and increased insulin expression. FGF2, known to be involved in pancreatic development [32,33], was identified as a frontrunner for further investigation. While MIN6 viability appeared insensitive to substrate composition, the limited time period of culture may have been insufficient to elucidate differences in long term viability. Col IV combinations ranked highly for both mouse and human islet attachment. These proteins are abundant in the islet basement membrane of both species, which is damaged during islet isolation [34,35]. We note that islet adhesion is more challenging to study using the array format, due to the weaker adhesion of aggregates to the substrate than single cells. In its current permutation, the microarray platform is also limited to be the study of biomolecules and markers localized to the cell population present at each microspot. However, segregation of test candidates in individual culture environments would allow secreted proteins, such as insulin, to be probed [36].

The utility of the ECM microarray platform extends beyond the specific application of islet attachment. Although this study documents the ability to profile adhesion patterns, cells bound to the arrays can be kept in culture for multiple days to monitor long-term responses to ECM such as cell death, proliferation and alterations in gene or protein expression. Overall, the ECM microarrays will enhance our ability to study a host of questions as they pertain to both basic biological and clinical settings.

## 5. Conclusions

We have developed a cell microarray platform and an assay protocol for accurate and sensitive analysis of differential MIN6 cell and islet population attachment to biomaterial surfaces in a high throughput manner while using minimum amounts of adhesive substrate and cells. We demonstrated the power of this approach in defining the substrate requirements that support adhesion of islets and islet derived cells, and then investigated the influence of these substrates on viability and insulin expression using MIN6 cells as a model for the β cell population within islets. We anticipate that the findings from the microarray study will enable the engineering of protein coatings on scaffold materials for β cell or islet transplantation.

## Figures and Tables

**Table 1:** Combinatorial protein microarrays layout used for array synthesis. The following 11 proteins were used: Type I collagen (Col I), type II collagen (Col II), type III collagen (Col III), type IV collagen (Col IV), laminin (Ln), fibronectin (Fn), fibroblast growth factor 2 (FGF-2), insulin-like growth factor 2 (IGF-2), vascular endothelial growth factor (VEGF), exenatide (Exen) and vitronectin (Vn). For single proteins and binary combinations, Col I, Col II, Col III, Col IV, Ln and Fn were printed at a concentration of 100 µg/mL; FGF-2, IGF-2, VEGF and Exen at a concentration of 25 µg/mL; and Vn at 50 µg/mL. In ternary combinations, the first protein listed was printed as the major component, at the concentration listed above, the second and third listed components were printed at 2/3 the concentration of the first listed protein.

	1	2	3	4	5	6	7	8	9	10	11	12	13	14	15
A	Col I	Col I/ Ln	Col II/ Exen	Col IV/ VEGF	FGF-2/ GF-2	Col IV/ Col I/ FGF-2	Col IV/ Col III/ Fn	Col IV/ Fn/ VEGF	Ln/ Col I/ Col III	Ln/ Col II/ Exen	Ln/ Col IV/ Vn	Ln/ VEGF/ Vn	FGF-2/ Col II/ Fn	FGF-2/ Col IV/ IGF-2	Col IV/ IGF-2/ Exen
B	Col II	Col I/ Fn	Col II/ /Vn	Col IV/ Exen	FGF-2 / VEGF	Col IV/ Col I/ IGF-2	Col IV/ Col III/ FGF-2	Col IV/ Fn/ Exen	Ln/ Col I/ Col IV	Ln/ Col II/ Vn	Ln/ Fn/ FGF-2	Ln/ Exen/ Vn	FGF-2/ Col II/ IGF-2	FGF-2/ Col IV/ VEGF	Col IV/ IGF-2/ Vn
C	Col III	Col I/ FGF-2	Col III/ Col IV	Col IV/ Vn	FGF-2/ Exen	Col IV/ Col I/ VEGF	Col IV/ Col III/ IGF-2	Col IV/ Fn/ Vn	Ln/ Col I / Fn	Ln/ Col III/ Col IV	Ln/ Fn / IGF-2	FGF-2/ Col I/ Col II	FGF-2/ Col II/ VEGF	FGF-2/ Col IV/ Exen	Col IV/ VEGF/ Exen
D	Col IV	Col I/ IGF-2	Col III/ Ln	Ln/ Fn	FGF-2/ Vn	Col IV/ Col I/ Exen	Col IV/ Col III/ VEGF	Col IV/ FGF-2/ IGF-2	Ln/ Col I/ FGF-2	Ln/ Col III/ Fn	Ln/ Fn VEGF	FGF-2/ Col I/ Col III	FGF-2/ Col II/ Exen	FGF-2/ Col IV/ Vn	Col IV/ VEGF/ Vn
E	Ln	Col I/ VEGF	Col III/ Fn	Ln/ FGF-2	IGF-2/ VEGF	Col IV/ Col I/ Vn	Col IV/ Col III/ Exen	Col IV/ FGF-2/ VEGF	Ln/ Col I / IGF-2	Ln/ Col III/ FGF-2	Ln/ Fn/ Exen	FGF-2/ Col I/ Col IV	FGF-2/ Col II/ Vn	FGF-2/ L / Fn	Col IV/ Exen/ Vn
F	Fn	Col I/ Exen	Col III/ FGF-2	Ln/ IGF-2	IGF-2/ Exen	Col IV/ Col II/ Col III	Col IV/ Col III/ Vn	Col IV/ FGF-2/ Exen	Ln/ Col I/ VEGF	Ln/ Col III/ IGF-2	Ln/ Fn/ Vn	FGF-2/ Col I/ Ln	FGF-2/ Col III/ Col IV	FGF-2/ Ln/ IGF-2	
G	FGF-2	Col I/ Vn	Col III/ IGF-2	Ln/ VEGF	IGF-2/ Vn	Col IV/ Col II/ Ln	Col IV/ Ln/ Fn	Col IV/ FGF-2/ Vn	Ln/ Col I/ Exen	Ln/ Col III/ VEGF	Ln/ FGF-2/ IGF-2	FGF-2/ Col I/ Fn	FGF-2/ Col III/ Ln	FGF-2/ Ln/ VEGF	
H	IGF-2	Col II/ Col III	Col III/ VEGF	Ln/ Exen	VEGF/ Exen	Col IV/ Col II/ Fn	Col IV/ Ln/ FGF-2	Col I/ IGF-2/ VEGF	Ln/ Col I/ Vn	Ln/ Col III/ Exen	Ln/ FGF-2/ VEGF	FGF-2/ Col I/ IGF-2	FGF-2/ Col III/ Fn	FGF-2/ Ln/ Exen	
I	VEGF	Col II/ Col IV	Col III/ Exen	Ln/ Vn	VEGF/ Vn	Col IV/ Col II/ FGF-2	Col IV/ Ln/ IGF-2	Col I/ IGF-2/ Exen	Ln/ Col II/ Col III	Ln/ Col III/ Vn	Ln/ FGF-2/ Exen	FGF-2/ Col I/ VEGF	FGF-2/ Col III/ IGF-2	FGF-2/ Ln / Vn	
J	Exen	Col II/ Ln	Col III/ Vn	Fn/ FGF-2	Exen/ Vn	Col IV/ Col II/ IGF-2	Col IV/ Ln/ VEGF	Col I/ IGF-2/ Vn	Ln/ Col II/ Col IV	Ln/ Col IV/ Fn	Ln/ FGF-2/ Vn	FGF-2/ Col I/ Exen	FGF-2/ Col III/ VEGF	FGF-2/ Fn/ IGF-2	
K	Vn	Col II/ Fn	Col IV/ Ln	Fn/ IGF-2	Col IV/ Col I Col II	Col IV/ Col II/ VEGF	Col IV/ Ln/ Exen	Col IV/ EGF/ Exen	Ln/ Col II/ Fn	Ln/ Col IV/ FGF-2	Ln/ IGF-2/ VEGF	FGF-2/ Col I/ Vn	FGF-2/ Col III/ Exen	FGF-2/ Fn VEGF	
L	Col I/ Col II	Col II/ FGF-2	Col IV/ Fn	Fn/ VEGF	Col IV/ Col I/ Col III	Col IV/ Col II/ Exen	Col IV/ Ln/ Vn	Col I/ VEGF/ Vn	Ln/ Col II/ FGF-2	Ln// Col IV/ IGF-2	Ln/ IGF-2/ Exen	FGF-2/ Col II/ Col III	FGF-2/ Col III/ Vn	FGF-2/ Fn/ Exen	
M	Col I/ Col III	Col II/ IGF-2	Col IV/ FGF-2	Fn/ Exen	Col IV/ Col I/ Ln	Col IV/ Col II/ Vn	Col IV/ Fn/ FGF-2	Col I/ Exen/ Vn	Ln/ Col II / IGF-2	Ln/ Col IV/ VEGF	Ln/ IGF-2/ Vn	FGF-2/ Col II/ Col IV	FGF-2/ Col IV/ Ln	FGF-2/ Fn/ Vn	
N	Col I/ Col IV	Col II/ VEGF	Col I/ IGF-2	Fn/ Vn	Col IV/ Col I/ Fn	Col IV/ Col III/ Ln	Col IV/ Fn/ IGF-2	Ln/ Col I/ Col II	Ln/ Col II/ VEGF	Ln/ Col IV/ Exen	Ln/ VEGF/ Exen	FGF-2/ Col II/ Ln	FGF-2/ Col IV Fn	Col IV/ IGF-2/ VEGF	

**Table 2:** Lead candidate combinations for MIN6 adhesion and growth, identified using the protein microarray.

Well ID	Formulation	Cells Attached Per Spot
D14	FGF-2/Col IV/Vn	168 ± 11.7
D11	Ln/Fn/VEGF	154 ± 6.39
N7	Col IV/Fn/IGF-2	153 ± 12.4
D12	FGF-2/Col I/Col III	152 ± 10.0
L7	Col IV/Ln/Vn	152 ± 10.5
L13	FGF-2/Col III/Vn	151 ± 9.82
F11	Ln/Fn/Vn	151 ± 11.0
L4	Fn/VEGF	145 ± 21.5
N4	Fn/Vn	138 ± 11.3
K4	Fn/IGF-2	131 ± 16.6
M11	Ln/IGF-2/Vn	126 ± 7.63
E14	FGF-2/Ln/Fn	125 ± 22.1
F5	IGF-2/Exen	125 ± 23.7
B5	FGF-2/VEGF	122 ± 19.1
J4	Fn/FGF-2	120 ± 9.91
N13	FGF-2/Col IV/Fn	117 ± 7.94
K1	Vn	115 ± 7.94
M2	Col II/IGF-2	113 ± 11.3
E13	FGF-2/Col II/Vn	113 ± 22.9
C13	FGF-2/Col II/VEGF	112 ± 11.72

## Supplementary Materials

The supplementary materials are available online at http://www.mdpi.com/2076-3905/5/3/21/s1.

## Abbreviations

The following abbreviations are used in this manuscript:

ECM	Extracellular matrix
HMDSO	Hexamethyldisiloxane
AGE	Allyl glycidyl ether
Col I	Type I collagen
Col II	Type II collagen
Col III	Type III collagen
Col IV	Type IV collagen
Ln	Laminin 111
Fn	Fibronectin
FGF-2	Fibroblast growth factor 2
IGF-2	Insulin-like growth factor 2
VEGF	Vascular endothelial growth factor
Exen	Exenatide
Vn	Vitronectin
TRITC	Tetramethylrhodamine

## References

[1] NCDRF Collaboration (2016). Worldwide trends in diabetes since 1980: A pooled analysis of 751 population-based studies with 4.4 million participants. Lancet.

[2] Roep B.O., Peakman M. (2012). Antigen targets of type 1 diabetes autoimmunity. Cold Spring Harb. Perspect. Med..

[3] Buchanan T.A. (2003). Pancreatic β-cell loss and preservation in type 2 diabetes. Clin. Ther..

[4] Duran-Salgado M.B., Rubio-Guerra A.F. (2014). Diabetic nephropathy and inflammation. World J. Diabetes.

[5] American Diabetes Association (2014). Diagnosis and classification of diabetes mellitus. Diabetes Care.

[6] Goland R., Egli D. (2014). Stem cell-derived β cells for treatment of type 1 diabetes?. EBioMedicine.

[7] Shapiro A.J., Ryan E.A., Lakey J.R. (2001). Islet cell transplantation. Lancet.

[8] Halban P.A., German M.S., Kahn S.E., Weir G.C. (2010). Current status of islet cell replacement and regeneration therapy. J. Clin. Endocrinol. Metab..

[9] Barton F.B., Rickels M.R., Alejandro R., Hering B.J., Wease S., Naziruddin B., Oberholzer J., Odorico J.S., Garfinkel M.R., Levy M. (2012). Improvement in outcomes of clinical islet transplantation: 1999–2010. Diabetes Care.

[10] Fischbach G.D., Fischbach R.L. (2004). Stem cells: Science, policy, and ethics. J. Clin. Investig..

[11] Xing Q., Vogt C., Leong K.W., Zhao F. (2014). Highly aligned nanofibrous scaffold derived from decellularized human fibroblasts. Adv. Funct. Mater..

[12] Riopel M., Wang R. (2014). Collagen matrix support of pancreatic islet survival and function. Front. Biosci..

[13] Mirmalek-Sani S.-H., Orlando G., McQuilling J.P., Pareta R., Mack D.L., Salvatori M., Farney A.C., Stratta R.J., Atala A., Opara E.C. (2013). Porcine pancreas extracellular matrix as a platform for endocrine pancreas bioengineering. Biomaterials.

[14] Goh S.-K., Bertera S., Olsen P., Candiello J.E., Halfter W., Uechi G., Balasubramani M., Johnson S.A., Sicari B.M., Kollar E. (2013). Perfusion-decellularized pancreas as a natural 3D scaffold for pancreatic tissue and whole organ engineering. Biomaterials.

[15] Roca-Cusachs P., Sunyer R., Trepat X. (2013). Mechanical guidance of cell migration: Lessons from chemotaxis. Curr. Opin. Cell Biol..

[16] Llacua A., de Haan B.J., Smink S.A., Smink A.M., de Vos P. (2016). Extracellular matrix components supporting human islet function in alginate based immunoprotective microcapsules for treatment of diabetes. J. Biomed. Mater. Res. A.

[17] Stendahl J.C., Kaufman D.B., Stupp S.I. (2009). Extracellular matrix in pancreatic islets: Relevance to scaffold design and transplantation. Cell Transplant..

[18] Bogdani M., Korpos E., Simeonovic C.J., Parish C.R., Sorokin L., Wight T.N. (2014). Extracellular matrix components in the pathogenesis of type 1 diabetes. Curr. Diabetes Rep..

[19] Weber L.M., Hayda K.N., Anseth K.S. (2008). Cell-matrix interactions improve β-cell survival and insulin secretion in three-dimensional culture. Tissue Eng. Part. A.

[20] Mei Y., Saha K., Bogatyrev S.R., Yang J., Hook A.L., Kalcioglu Z.I., Cho S.-W., Mitalipova M., Pyzocha N., Rojas F. (2010). Combinatorial development of biomaterials for clonal growth of human pluripotent stem cells. Nat. Mater..

[21] Hook A.L., Thissen H., Voelcker N.H. (2006). Surface manipulation of biomolecules for cell microarray applications. Trends Biotechnol..

[22] Hook A.L., Anderson D.G., Langer R., Williams P., Davies M.C., Alexander M.R. (2010). High throughput methods applied in biomaterial development and discovery. Biomaterials.

[23] Hook A.L., Thissen H., Voelcker N.H. (2009). Advanced substrate fabrication for cell microarrays. Biomacromolecules.

[24] Anglin E., Davey R., Herrid M., Hope S., Kurkuri M., Pasic P., Hor M., Fenech M., Thissen H., Voelcker N.H. (2010). Cell microarrays for the screening of factors that allow the enrichment of bovine testicular cells. Cytom. A.

[25] Kurkuri M.D., Driever C., Johnson G., McFarland G., Thissen H., Voelcker N.H. (2009). Multifunctional polymer coatings for cell microarray applications. Biomacromolecules.

[26] Coad B.R., Jasieniak M., Griesser S.S., Griesser H.J. (2013). Controlled covalent surface immobilisation of proteins and peptides using plasma methods. Surf. Coat. Tech..

[27] Rasi Ghaemi S., Harding F., Delalat B., Vasani R., Voelcker N.H. (2013). Surface engineering for long-term culturing of mesenchymal stem cell microarrays. Biomacromolecules.

[28] Nikolova G., Jabs N., Konstantinova I., Domogatskaya A., Tryggvason K., Sorokin L., Fässler R., Gu G., Gerber H.-P., Ferrara N. (2006). The vascular basement membrane: A niche for insulin gene expression and β cell proliferation. Dev. Cell.

[29] Daoud J.T., Petropavlovskaia M.S., Patapas J.M., Degrandpre C.E., Diraddo R.W., Rosenberg L., Tabrizian M. (2011). Long-term in vitro human pancreatic islet culture using three-dimensional microfabricated scaffolds. Biomaterials.

[30] Daoud J., Petropavlovskaia M., Rosenberg L., Tabrizian M. (2010). The effect of extracellular matrix components on the preservation of human islet function in vitro. Biomaterials.

[31] Durr J., Goodman S., Potocnik A., von der Mark H., von der Mark K. (1993). Localization of β 1-integrins in human cartilage and their role in chondrocyte adhesion to collagen and fibronectin. Exp. Cell Res..

[32] Hardikar A.A., Marcus-Samuels B., Geras-Raaka E., Raaka B.M., Gershengorn M.C. (2003). Human pancreatic precursor cells secrete FGF2 to stimulate clustering into hormone-expressing islet-like cell aggregates. Proc. Natl. Acad. Sci. USA.

[33] Inchovska M., Ogneva V., Martinova Y. (2006). Role of FGF1, FGF2 and FGF7 in the development of the pancreas from control and streptozotocin-treated hamsters. Cell Prolif..

[34] Irving-Rodgers H.F., Choong F.J., Hummitzsch K., Parish C.R., Rodgers R.J., Simeonovic C.J. (2014). Pancreatic islet basement membrane loss and remodeling after mouse islet isolation and transplantation: Impact for allograft rejection. Cell Transplant..

[35] Wang R., Rosenberg L. (1999). Maintenance of β-cell function and survival following islet isolation requires re-establishment of the islet-matrix relationship. J. Endocrinol..

[36] Flaim C.J., Teng D., Chien S., Bhatia S.N. (2008). Combinatorial signaling microenvironments for studying stem cell fate. Stem Cells Dev..

