# Supplementary material for: A Combinatorial Protein Microarray for Probing Materials Interaction with Pancreatic Islet Cell Populations
Source: Microarrays (Basel). 2016 Aug 10;5(3):21. doi: 10.3390/microarrays5030021 (PMC5040968; doi:10.3390/microarrays5030021)
Supplement: Supplementary file 1 [file microarrays-05-00021-s001.pdf]

Supplementary Materials: A Combinatorial Protein Microarray for Probing Materials Interaction with Pancreatic Islet Cell Populations

Bahman Delalat, Darling Rojas-Canales, Soraya Rasi Ghaemi, Michaela Waibel, Frances J. Harding, Daniella Penko, Christopher J. Drogemuller, Thomas Loudovaris, Patrick T.H. Coates and Nicolas H. Voelcker

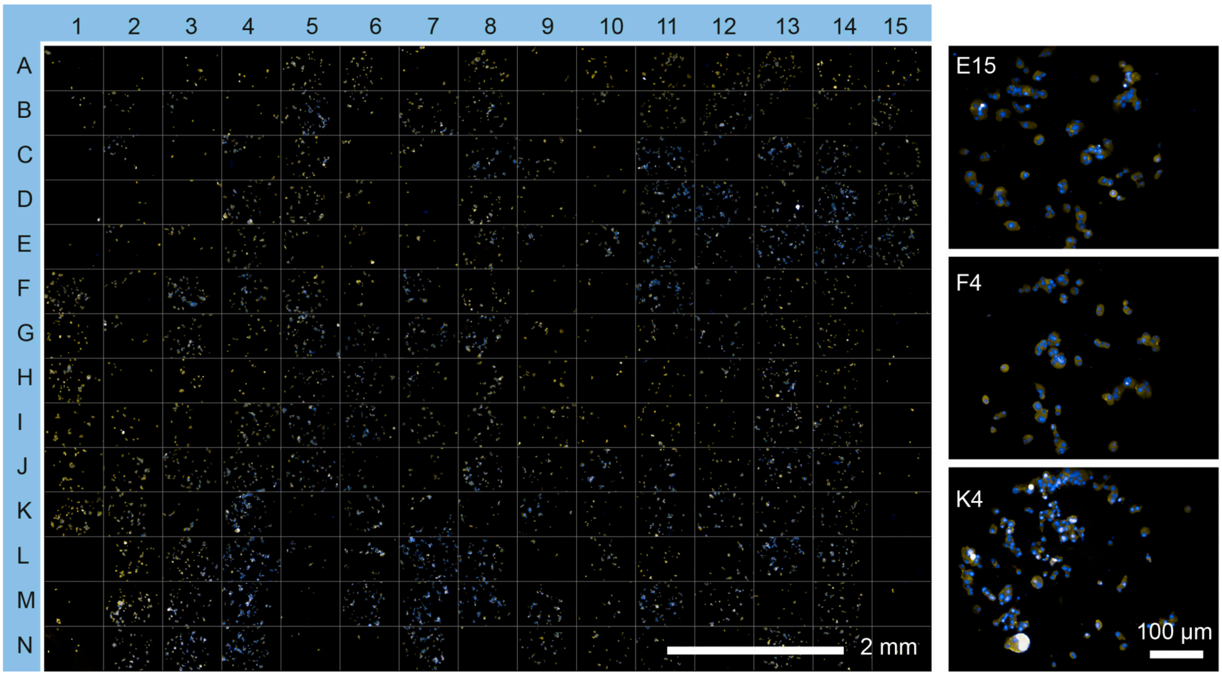

Figure S1. Attachment of MIN6 cells on the cell microarray. MIN6 cells are stained with TRITC-labeled phallodin (yellow) and counterstained with Hoechst 33242 (blue). Insets show selected microspots at higher resolution.

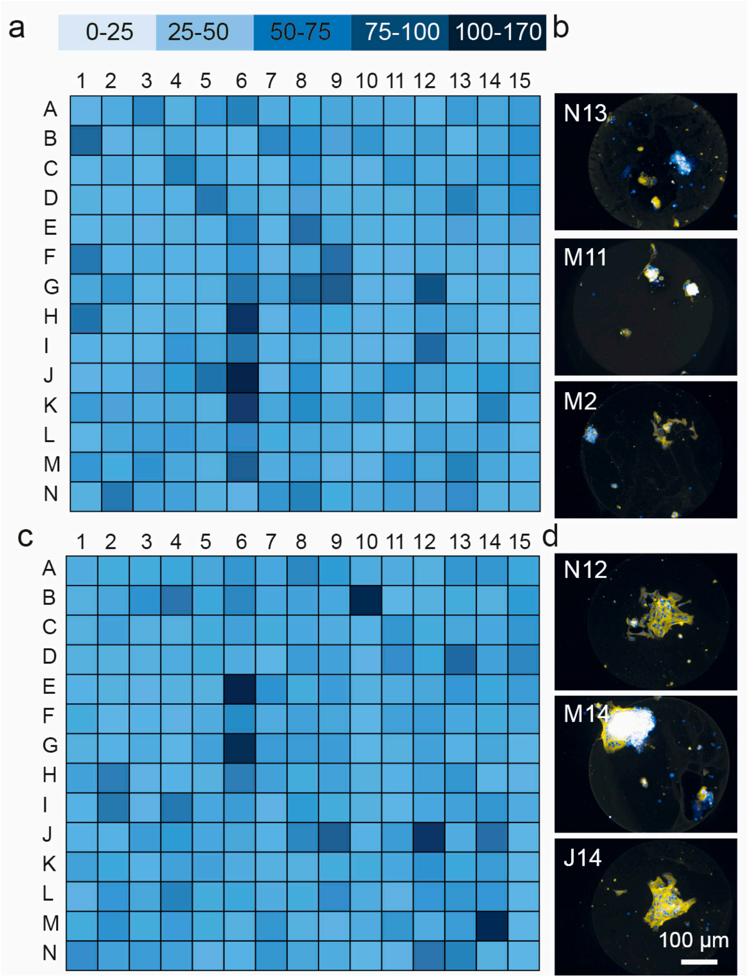

Figure S2. Quantification of islets adhesion to the spots on protein microarray: (**a**) map of cell attachment primary mouse islets; (**b**) representative images of primary mouse islets adhering to selected protein microarray (N13, M11 and M2); (**c**) map of cell attachment primary human islets for all of the 201 protein combinations in the array; and (**d**) representative images of primary human islets adhering to selected protein spots (N12, M14 and J14). *N* = 3.

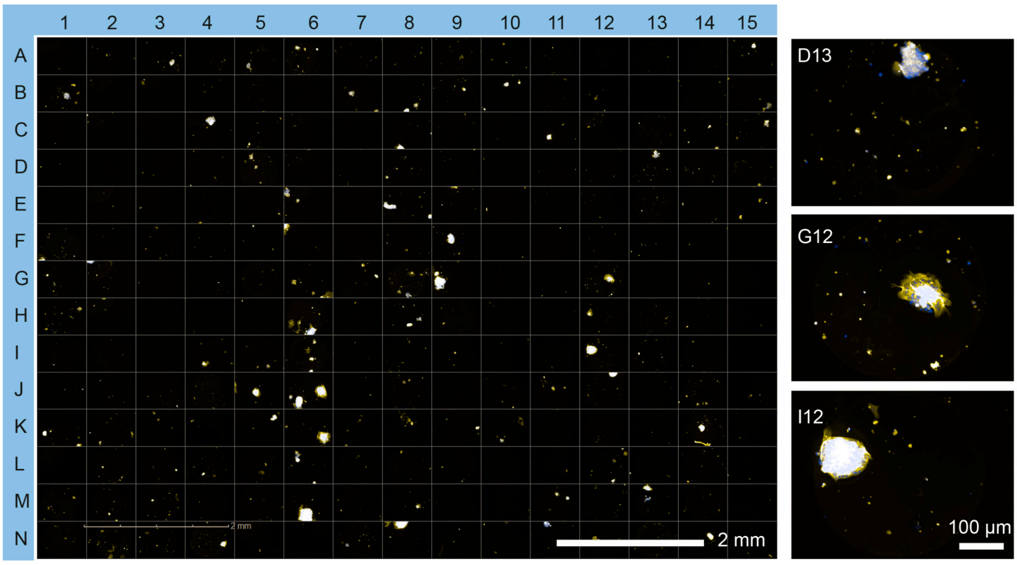

Figure S3. Attachment of mouse islets on the cell microarray. Mouse islets are stained with TRITC-labeled phalloidin (yellow) and counterstained with Hoechst 33242 (blue). Insets show selected microspots at higher resolution.

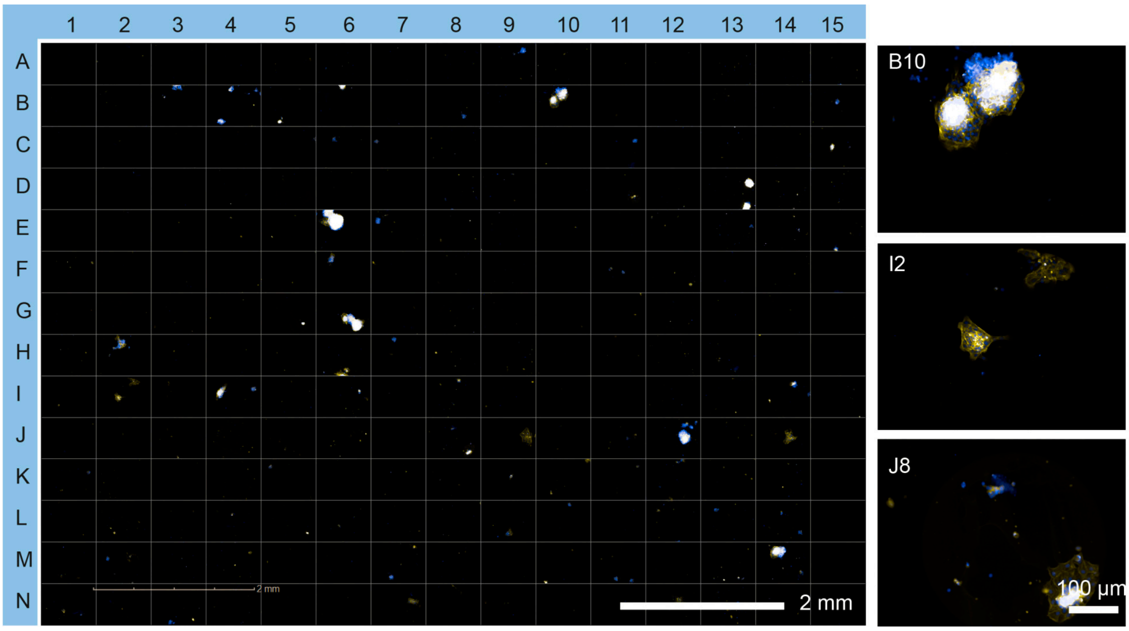

Figure S4. Attachment of human islets on the cell microarray. Mouse islets are stained with TRITC-labeled phalloidin (yellow) and counterstained with Hoechst 33242 (blue). Insets show selected microspots at higher resolution.

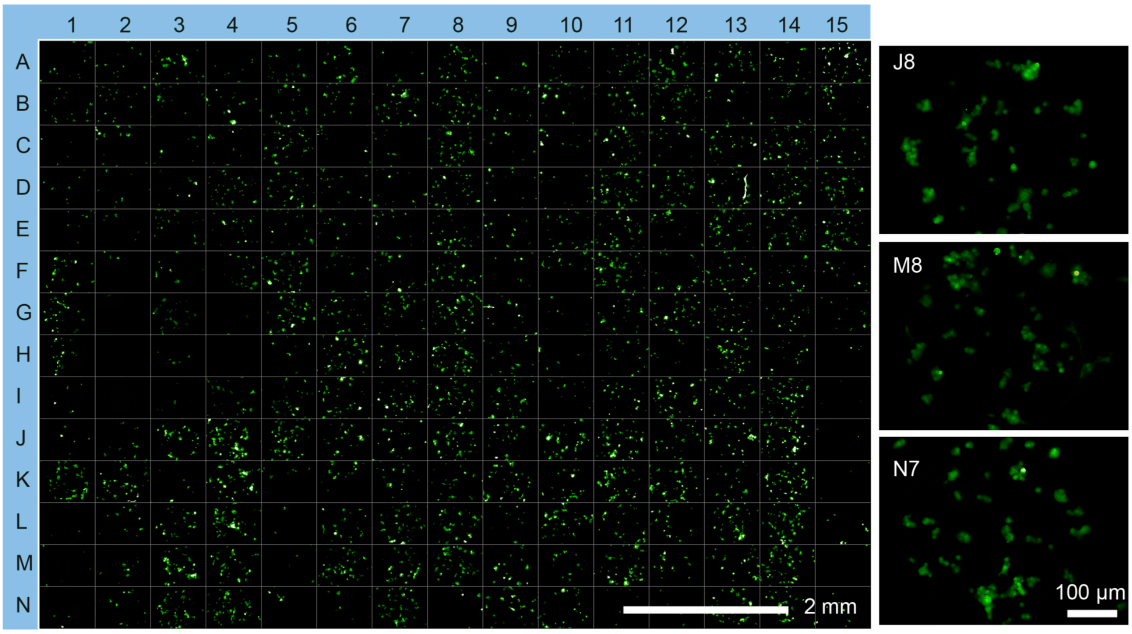

Figure S5. Cell viability of MIN6 cells on the cell microarray. Viable cells are stained in green (fluorescein diacetate), and the dead cells were stained in red (propidium iodide). Insets show selected microspots at higher resolution.

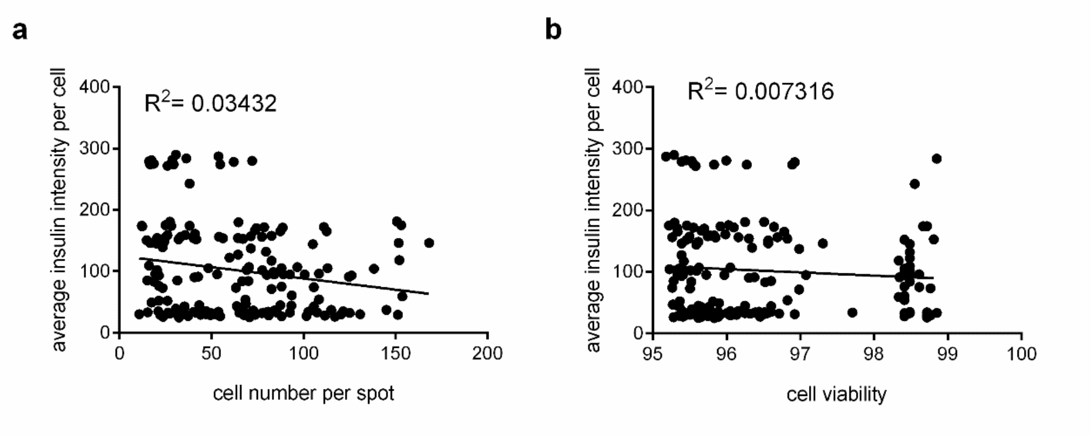

Figure S6. Linear regression of: (**a**) average insulin intensity per cell vs. cell number per spot; and (**b**) average insulin intensity per cell vs. cell viability for MIN6 (data sets shown in Figures 3, 4, and Figure S5).
